# Precise Deposition of Carbon Nanotube Bundles by Inkjet-Printing on a CMOS-Compatible Platform

**DOI:** 10.3390/ma15144935

**Published:** 2022-07-15

**Authors:** Rohitkumar Shailendra Singh, Katsuyuki Takagi, Toru Aoki, Jong Hyun Moon, Yoichiro Neo, Futoshi Iwata, Hidenori Mimura, Daniel Moraru

**Affiliations:** 1 Graduate School of Science and Technology, Shizuoka University, 3-5-1 Johoku, Naka-ku, Hamamatsu 432-8011, Japan; 2 Research Institute of Electronics, Shizuoka University, 3-5-1 Johoku, Naka-ku, Hamamatsu 432-8011, Japan; rohitkumar.singh.19@shizuoka.ac.jp (R.S.S.); takagi.katsuyuki@shizuoka.ac.jp (K.T.); aoki.toru@shizuoka.ac.jp (T.A.); moon.jonghyun@shizuoka.ac.jp (J.H.M.); neo.yoichiro@shizuoka.ac.jp (Y.N.); iwata.futoshi@shizuoka.ac.jp (F.I.); mimura.hidenori@shizuoka.ac.jp (H.M.)

**Keywords:** carbon nanotube field-effect transistors, nano-transistor, CMOS-compatibility, AFM manipulation, inkjet printing

## Abstract

Carbon nanotubes (CNTs) are ultimately small structures, attractive for future nanoelectronics. CNT-bundles on Si nanostructures can offer an alternative pathway to build hybrid CMOS-compatible devices. To develop a simple method of using such CNT-bundles as transistor channels, we fabricated semiconductor single-walled CNT field-effect transistors using inkjet printing on a CMOS-compatible platform. We investigated a method of producing stable CNT solutions without surfactants, allowing for CNT debundling and dispersion. An inkjet-printing system disperses CNT-networks with ultimately low density (down to discrete CNT-bundles) in Al source-drain gaps of transistors. Despite the small number of networks and random positions, such CNT-bundles provide paths to the flow current. For enhanced controllability, we also demonstrate the manipulation of CNT-networks using an AFM technique.

## 1. Introduction

The miniaturization of electronics has brought the dimensions of the key devices, Si transistors, well into the nanoscale [1] where several fundamental challenges have emerged. As an alternative, carbon nanotubes (CNTs) such as semiconductor single-walled CNTs (SW-CNTs) have been considered for future electronics specifically because of their outstanding transport properties [2,3,4,5], naturally nanoscale dimensions [6,7], and potential flexibility [8,9,10,11,12]. By implementing several techniques for the deposition and manipulation of SW-CNTs on complementary metal-oxide-semiconductor (CMOS) platforms, hybrid CNT/Si devices can be evaluated.

SW-CNTs are thus an appealing candidate for fabricating hybrid nano-transistors. Researchers have attempted to fabricate such CNT-based transistors using direct growth of the CNTs on a substrate, but the main issue with this method is controlling the chirality of the CNTs, which can easily change with small deviations in the dimensions of the CNTs. Depending on the chirality, SW-CNTs can exhibit either metallic or semiconducting behavior [8,13,14,15]. Any metallic CNTs introduced by the direct-growth method degrade the transistor operation. In addition, the control of the density of CNTs and of the assembly/position of CNTs in the device remain significant challenges in fabricating the CNT field-effect transistors (CNT-FETs). Inkjet-printing technology is promising in this sense because patterns can be generated at a specific location by controlling the CNT-ink droplet size, without material waste, drastically reducing the material dispersion and production costs [16]. In comparison to other CNT deposition methods such as CVD growth, dry filtration, spin coating, or drop coating on large areas, inkjet-printing holds promise as a method suitable for device integration as it offers the targeted deposition of CNTs, eventually adaptable for industrial-scale production. However, typical inkjet-printing approaches aim to deposit CNT films, rather than individual CNTs, on various surfaces, using relatively large nozzles or in large gaps.

In this paper, we investigated the fabrication and electrical characteristics of the CNT-FETs with discrete CNT-bundles as channels, dispersed using inkjet-printing technology to ultimately achieve low density and highly precise location (using relatively small inkjet nozzles). In addition, the deposition of low-density CNTs (or CNT-bundles) is carried out on the Si/SiO_2_ surface between the Al source and drain, within gaps of only a few hundred nm, on CMOS-compatible platforms. In most other works that used similar solutions, the density was significantly higher to form thick CNT-films [17,18,19,20,21], typically on substrates different from SiO_2_, except for a few reports [22,23,24]. In a report of the inkjet printing of CNTs on CMOS-compatible substrates, even at low density [23], the gap between the source and drain is on the order of mm and no Al electrodes were used (instead, multiple inkjet printings were used to form electrodes). In this work, CNT-networks were also deposited by inkjet printing from similar solutions using dimethylformamide (DMF) as a solvent, which has some advantages compared to other solvents such as N-methyl-2-pyrrolidone (NMP) [24], and has been demonstrated to be a better solvent than water-based functionalization [25,26,27,28,29]. Thus, our objective was to demonstrate inkjet printing of *low-density CNT-networks* on *highly CMOS-compatible platforms* (including Al electrodes) in relatively small source-drain gaps of about 1 µm.

As a first step, the homogeneity and dispersion conditions of the SW-CNTs were optimized by long-time sonication. The step of obtaining a homogeneous solution is critical since van der Waals forces between CNTs can lead to the formation of large bundles or agglomerates, which would make the interpretation of the transport characteristics more complex and challenging [16]. A second stage is to analyze the results of different inkjet-printing conditions by field emission scanning electron microscope (FE-SEM) measurements. The preliminary results are discussed in some detail in the Supplementary Materials, Figure S1.

## 2. Materials and Methods

**Figure 1:**
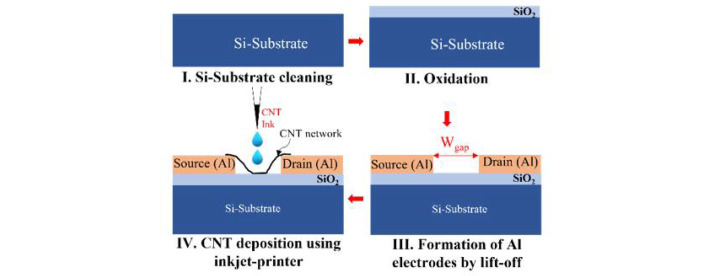
Process flow of the CNT-FET fabrication with inkjet-printed CNTs deposited in between Al source and drain. (I) Preparing the silicon wafer by cleaning. (II) Dry oxidation for the growth of a SiO_2_ layer (approx. thickness of 30 nm). (III) Preparing by a lift-off process the source and drain electrodes (defined in Al, approx. thickness of 70 nm) with gaps of different widths, W_gap_. (IV) CNT printing for deposition in between the source and drain electrodes.

Figure 1 illustrates the details of the fabrication process of the CNT-FETs using inkjet-printing to place CNT-networks at a relatively low density between source and drain. Highly purified, pre-separated, 99.9% purity semiconductor-CNTs (NanoIntegris) were utilized to prepare CNT-ink for the inkjet printing process. First, CNT-FET fabrication was initiated on a *p*-type silicon (Si) substrate (≈10^15^ cm^−3^) that can work as a back gate, with a 30-nm-thick silicon dioxide (SiO_2_) layer grown by dry thermal oxidation. Next, the source and drain regions were patterned by an electron beam (EB) lithography technique, and then deposition of the metal layer (here, aluminum, Al) was carried out by an evaporation method. Finally, the lift-off process allowed the definition of the gap between source and drain, with its width (W_gap_) as a parameter.

At the next stage, we prepared the CNT-ink for inkjet printing. CNTs tend to form dense clumps, requiring a significant amount of coercion to disperse them effectively [30]. For this reason, we used long-time, high-power sonication using a bath-type sonicator at 100 W and 38 kHz.

It was observed that DMF, a dipolar organic solvent, is an effective solvent for producing CNT solutions without any surfactants. The combination of the excellent solvency properties of DMF and inkjet printing allows the suitable dispersion of CNTs without a surfactant, which is usually necessary to fabricate transistors without degrading the performance. Since the CNT source material was in the thick-film condition initially, the solutions in this study were ultrasonicated for about 4 h immediately before inkjet printing, which is a longer time than typically reported, for the specific purpose of obtaining good homogeneity of the solution. Although there are reports that damage to the structure of the CNTs may occur after long-time sonication [31,32], it was not expected to have a significant effect in our experiments where CNT-bundles still remained in the solution, even after such a long-time processing.

For this work, we used low concentration (≈1 µg/mL) CNT-solutions, so we obtained very few paths of CNT-networks in between the Al source and drain. In contrast to the target of this work, which was low-density CNT-networks, if a higher density is required, it can be achieved by multiple printing or a higher concentration of the CNT-solution itself.

After preparing the homogeneous solution as a CNT ink, we dispersed it at precise locations using an inkjet-printing system (SIJ-S050, SIJTechnology Inc., Tsukuba, Japan), as shown in Supplementary Materials Figure S2. Figure 1 also presents a schematic illustration of the CNT-FET fabrication processes (panels I–III), with inkjet-printed CNT-network(s) placed between source and drain (panel IV). Droplets of CNT-ink were dispersed by inkjet-printing in the sub-nL range through a piezoelectric nozzle using a sine wave at a frequency of 500 Hz and an optimized amplitude of 300 V. The droplet may take several minutes to completely evaporate at room temperature. This may also lead to re-agglomeration; therefore, printing at a higher substrate temperature or introducing tailored drying conditions must be taken into consideration. However, at present, to understand the challenges posed by the inkjet printing under regular conditions, all samples were allowed to dry in ambient atmosphere.

We used a 5-µm-diameter nozzle to disperse the CNT-networks on the samples. The size of the nozzle was also smaller than typically reported because the aim was to control the deposition location as much as possible. The CNTs were dispersed at various positions, as well as in different gaps. The CNT dimensions were on the order of 1 µm in length and 1.2–1.7 nm in diameter. However, even in the best dispersion conditions, some bundles still exist in the solution due to the van der Waals forces. Sometimes, larger agglomerates of CNTs lead to clogging of the inkjet nozzle. Therefore, we used a low-density CNT-ink while printing to also lower the probability of nozzle clogging.

## 3. Results

**Figure 2:**
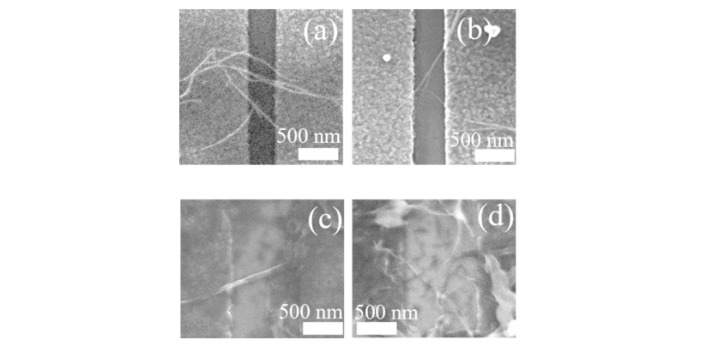
FE-SEM images of CNT-networks deposited by inkjet printing at low density from a 99.9% semiconductor-CNT solution after 4 h of ultrasonication on various source-drain gaps. (**a**,**b**) FE-SEM images of two different paths in a CNT-FET with a rectangular gap (designed gap, W_gap_ = 500 nm) between Al source and drain electrodes. (**c**,**d**) FE-SEM images of two different paths in a CNT-FET with a rectangular gap (designed gap, W_gap_ = 1000 nm) between Al source and drain electrodes.

Figure 2 shows examples of the CNT dispersion by inkjet printing on Al source and drain gaps with different designed dimensions (W_gap_ = 500 nm and 1000 nm). The coverage of the CNT-networks was low given the low concentration of the CNT-solution and long-time ultrasonication (about 4 h) before inkjet printing.

Figure 2a–d shows FE-SEM images of devices in which a few CNT-networks bridge the gap between the electrodes. It should be noted that out of the 28 devices of this type for which inkjet printing was performed under the same conditions, CNT-networks were successfully deposited between source and drain in 25 of them. In fact, there were several percolation CNT-networks visible in the FE-SEM images, most likely formed by bundles of CNTs.

**Figure 3:**
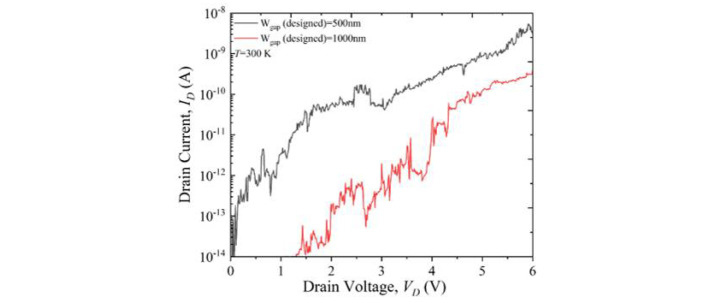
Room temperature *I_D_*–*V_D_* characteristics for two CNT-FETs from the same sample (black curve for a designed gap = 500 nm (FE-SEM images shown in Figure 2a,b) and red curve for a designed gap = 1000 nm (FE-SEM images shown in Figure 2c,d)).

Next, the electrical characteristics of the CNT-FETs were measured using a Keysight 4156C semiconductor precision parameter analyzer at room temperature (*T* = 300 K), as shown in Figure 3, and at low temperature (*T* = 8.5 K), as shown in Supplementary Figures S3 and S4 for devices with various gaps, under vacuum conditions.

These results revealed that *I_D_* flows through the channels containing CNT-networks for almost all the gaps, both at room temperature and at low temperature, as shown in Supplementary Figures S3 and S4. The critical difference between the two regimes of temperature was the shift to a much higher *V_D_* observed at low temperature, indicating the high resistivity of the devices. In order to explain the results, it should be mentioned that the CNTs were printed on top of the source and drain electrodes, so it is expected that the CNT/metal contact resistance might be high due to the intrinsic Schottky barrier and depends on the specific conditions of the deposition. In the present work, since CNT-bundles were deposited on the surface of the Al electrodes for the simplicity of fabrication and manipulation (as will be shown later), the mechanical connection between CNT-bundles and Al may also be relatively weak. Placing the CNT-bundles underneath the Al electrodes could solve the connection problem, but would make the fabrication more complex in terms of the lift-off process.

## 4. Discussion

Figure 3 shows the room temperature output *IV* characteristics for two devices from the same sample (black curve for a device with a designed gap = 500 nm (FE-SEM images shown in Figure 2a,b) and red curve for a device with a designed gap = 1000 nm (FE-SEM images shown in Figure 2c,d)). The transfer characteristics could not be measured because of the lack of optimization of the gate (in this case, the weakly-doped Si substrate). The output characteristics depend on the number of CNT-bundles connected from the source to the drain and on the distance between the electrodes (i.e., the dimensions of the source–drain gap). Both these factors work to enhance *I_D_* for the case of the device with a 500 nm designed gap compared to the device with a 1000 nm designed gap. Since the CNTs have an average length of 1 µm, some would be disconnected from either the source or the drain in larger gaps, but this would be beneficial to preserve only a small number of paths, which was the target of this work. In any case, in the present devices, it is likely that most of the current flows through CNT-bundles formed by multiple connected CNTs. However, due to the scarcity of CNT-bundles in both devices, the current is still relatively low and a large *V_D_* is needed to generate a significant amount of current. In addition, although the characteristics are generally reproducible in consecutive measurements, there are significant differences from device to device, even if fabricated under the same conditions.

The characteristics for the two devices shown here exhibited both abrupt changes in current, similar to random telegraph signals (RTS), suggesting the presence of electrical traps in the nearby environment. Since the CMOS platform on which the deposition was performed was formed by dry oxidation of the SiO_2_ layer in cleanroom conditions, followed by hydrogen annealing after completion of the electrodes, traps at the Si/SiO_2_ interface are unlikely to cause these RTS features. A more likely origin can be the interactions between different CNT-bundles mixed in small networks on top of the Al electrodes. The Schottky barrier created at the CNT/Al interface, as well as the relatively weak connection between the CNT-bundles and Al electrodes, may also play an important role in this behavior. It is likely that replacing Al with other metals such as Au or Ti [33], for instance, can allow a better condition for transport, but it would also deviate from our main purpose of developing such devices on a CMOS-compatible platform. Further analysis is needed to specifically reveal the mechanism of the electrostatic interactions between the neighboring CNT-bundles and between the CNT-bundles and Al electrodes. The alignment and separation of the CNT-bundles is also expected to minimize such RTS features.

Due to the low concentration of the CNT-solution and due to the remaining uncontrollability in the inkjet-printing process, there is sometimes a misalignment of the CNT-networks within the gap, as shown by other examples in Supplementary Figure S5. The degree of position accuracy depends on a combination of factors including the nozzle size (~5 µm), the lateral size of the gap between facing electrodes (for the cases such as shown in Supplementary Figure S5, only 3 µm), and the gap width (typically on the order of 1 µm). Out of the 27 devices with such smaller gaps that were printed, CNT-bundles were found bridging the shortest gap in 16 devices. Thus, it can be considered that the present positioning accuracy is on the order of 3 µm. In order to further control the arrangement of these CNT-bundles, specifically to interrupt some of them, one possible approach is to employ a high-speed AFM nano-manipulator coupled with a haptic device [34] to modify the CNT-network mechanically.

Further technical details about the AFM technique used in this work (in tapping mode and in air atmosphere) are provided in the last section of the Supplementary Materials. It can be expected that such a technique allows the disconnection (interruption) of some of the transport paths (CNT-bundles), so that a dominant path can be identified. In previous works, CNT manipulation and soldering was demonstrated and reported on a Si surface [35], while AFM nanomanipulation of ultrasonically dispersed and oxidized CNTs was also analyzed on a Si surface [36].

Here, to show a proof-of-concept capability in this study of AFM manipulation, CNT-networks were deposited from a N-methyl-2-pyrrolidone (NMP) solution (by pipette dropping, in this case) onto a glass or Si/SiO_2_ surface and then manipulated by the above-indicated method. First, we demonstrated this manipulation on a glass substrate, as a preliminary result, shown in Supplementary Figure S6. We focused, however, on the demonstration of such manipulation of the CNT-networks deposited on thermally-grown SiO_2_, which is a more challenging surface, but is more suitable for confirming the CMOS-compatibility for the manipulation process.

**Figure 4:**
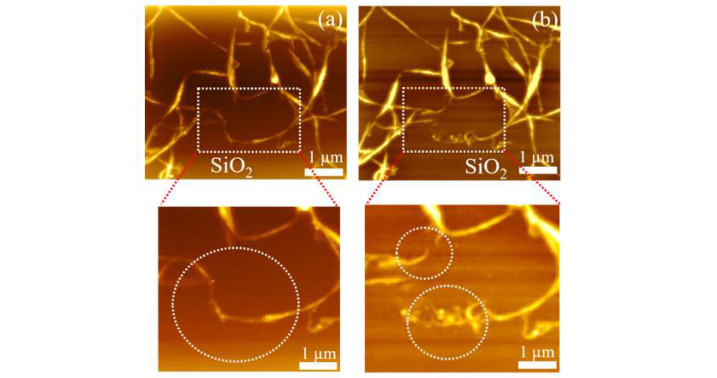
AFM images of a CNT-network deposited on a thermally-grown SiO_2_ surface from NMP solution before (**a**) and after (**b**) AFM manipulation (performed only in the area indicated by the dotted box). Zoom-in images are shown below for clarity, illustrating two different effects in the encircled area: distortion (interruption) of a CNT-bundle (lower area encircled in (**b**)) and shift of two branches of the CNT-network (upper area encircled in (**b**)).

Figure 4 shows the basic demonstrations of the modification of a CNT-network on the SiO_2_ surface when the AFM tip was moved within the area indicated by the dotted box. As shown in the bottom zoom-in images, the CNT-network was distorted only in the areas where manipulation was intended by repeated contact with the AFM tip (as marked in the lower part of Figure 4b), followed by a shift of two distinct branches toward each other (as marked in the upper part of the zoom-in image shown in Figure 4b). These effects can be observed more clearly by comparing the image in (a) (before AFM manipulation) and (b) (after AFM manipulation). It is expected that such manipulation is possible because the CNT-bundles observed in the CNT-network are likely formed by multiple CNTs that can be separated and adjusted in position by an AFM tip. Potentially, such manipulation can allow the optimization of CNT-networks.

Since the network shown here is comparable with the ones observed in between the Al source and drain electrodes (for example, as shown in the FE-SEM images of Figure 2), it is expected that this method can be adapted to manipulate (connect, disconnect, and/or align) the CNT-networks deposited in practical devices. This remains a target for future research, in which the thickness of the Al electrodes also has to be optimized for proper manipulation in the Si/SiO_2_/Al stack of a device.

## 5. Conclusions

We reported the fabrication of hybrid CNT-bundle-based transistors on a CMOS-compatible platform. The basic results are shown for the deposition of CNT-bundles without any surfactants on the Si/SiO_2_ surface in a few-hundred-nm gaps between Al source and drain electrodes, using inkjet printing for high localization. We used a low density CNT-network at the channel in order to decrease the probability of having a complex network with multiple pathways for current flow between source and drain electrodes. In addition, we reported a proof-of-concept capability for the manipulation of CNTs by an AFM technique. It is expected that further optimization can allow more precise control of CNTs in such systems, opening pathways for the development of hybrid nano-electronics.

## Data Availability

Data are available upon request.

## Supplementary Materials

The following supporting information can be downloaded at: https://www.mdpi.com/article/10.3390/ma15144935/s1, Figure S1: Optimization of the CNT solution; Figures S2–S4: Localized deposition using an inkjet-printing technique; Figures S5 and S6: Misalignment of the CNT-network and proof-of-concept manipulation.
