# Supplementary material for: Precise Deposition of Carbon Nanotube Bundles by Inkjet-Printing on a CMOS-Compatible Platform
Source: Materials (Basel). 2022 Jul 15;15(14):4935. doi: 10.3390/ma15144935 (PMC9323799; doi:10.3390/ma15144935)
Supplement: Supplementary file 1 [file materials-15-04935-s001.zip › materials-1802089-supplementary.pdf]

Supplementary Materials
Precise Deposition of Carbon Nanotube Bundles by Inkjet-Printing on a CMOS-compatible Platform

Rohitkumar Shailendra Singh,[1,2] Katsuyuki Takagi,[2] Toru Aoki,[1,2] Jong Hyun Moon,[2] Yoichiro Neo,[1,2] Futoshi Iwata,[1,2] Hidenori Mimura[1-2] and Daniel Moraru[1,2*]

[1] *Graduate School of Science and Technology, Shizuoka University, 3-5-1 Johoku, Naka-ku, Hamamatsu 432-8011, Japan*
[2] *Research Institute of Electronics, Shizuoka University, 3-5-1 Johoku, Naka-ku, Hamamatsu 432-8011, Japan*
*E-mail: moraru.daniel@shizuoka.ac.jp

Optimization of CNT solution:

The stability of a homogeneous CNT solution for inkjet printing is crucial for the time-efficient fabrication of CNT-FETs. We started to make a CNT ink using water-based CNT solutions, but an agglomeration problem prohibited the achievement of a highly stable solution. Therefore, the focus was shifted to make CNT solutions using organic solvent without surfactants. Throughout most of this work, we used dimethylformamide (DMF) solvent to prepare a homogeneous solution by long-time sonication, in order to avoid the agglomeration and disperse properly the CNTs. Figure S1 shows a comparison between CNT-solutions deposited by pipette dropping (without control), as shown in Fig. S1a, and deposited by inkjet printing, as shown in Fig. S1b.

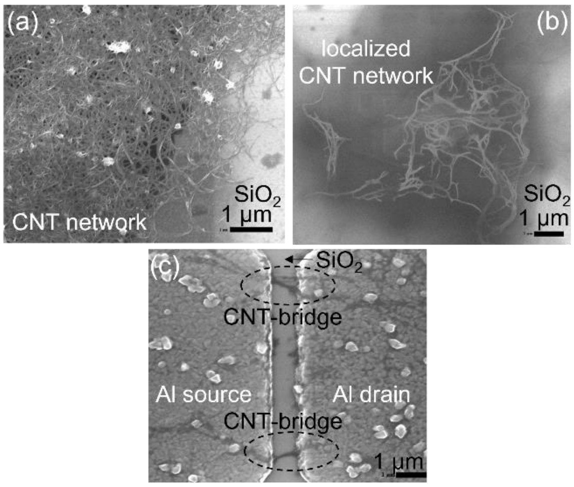

Figure S1. FE-SEM images of CNT-networks obtained from DMF-solution deposited in various configurations: (a) by pipette dropping on SiO_2; (b) by inkjet printing on SiO_2; (c) by inkjet printing in an Al source-drain gap, with a few CNT-bundles (marked as CNT-bridges) bridging the source and drain electrodes.

It can be seen that a complex network of CNTs is formed on a large area by pipette dropping, while a localized array of CNTs can be formed on an area with dimensions on the order of a few µm by inkjet printing. In both cases, the underlying surface is a thermally-grown SiO_2 layer. When deposition is properly done on a nanoscale gap between Al electrodes, several CNT-bridges can be observed (Fig. S1c).

Localized deposition using an inkjet-printing technique:

After confirming the solution stability, the deposition is carried out using an inkjet-printing system. Typically, the process for obtaining a homogeneous, stable CNT solution in DMF involves ultrasonication for about 4 hours (h) to avoid agglomeration problems and clogging of the nozzle. Figure S2 shows the inkjet-printing system (SIJ-S050, SIJTechnology inc.) before printing, displaying the way of alignment of the nozzle with the source-drain gap. The nozzle position can be thus localized (controlled) in a range of a few µm. Positioning accuracy depends on several factors, including nozzle size (~5 µm), lateral dimensions of source-drain gap (50 µm for the so-called rectangular-shaped gaps, but only 3 µm for the so-called trapezoidal-shaped gaps), and the source-drain gap (typically, on the order of 1 µm or less).

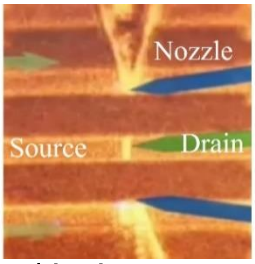

Figure S2. Alignment of the inkjet-printing system, with the nozzle (top) and reflection (bottom) allowing the positioning of the deposition in between source and drain electrodes. Markers allow the positioning within a range of a few µm, while the positioning accuracy due to several factors in our design is estimated to be about 3 µm.

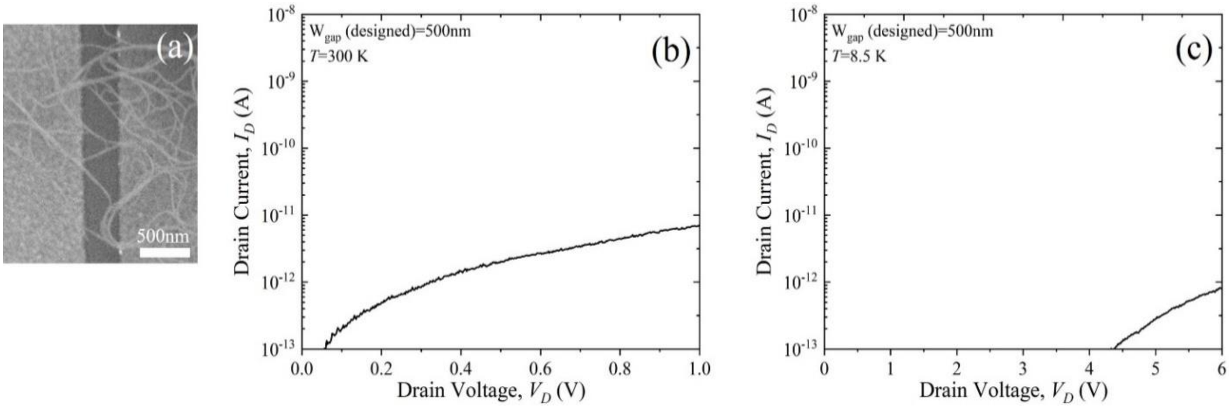

Figure S3. (a) FE-SEM image of a CNT bridge network deposited by inkjet printing in a gap (designed gap = 500 nm) between Al source and drain electrodes. (b) Room-temperature I_D–V_D characteristics for the same device. (c) Low-temperature (T = 8.5 K) I_D–V_D characteristics for the same device. Characteristics are measured in different V_D ranges as the onset voltage is largely shifted at low temperature.

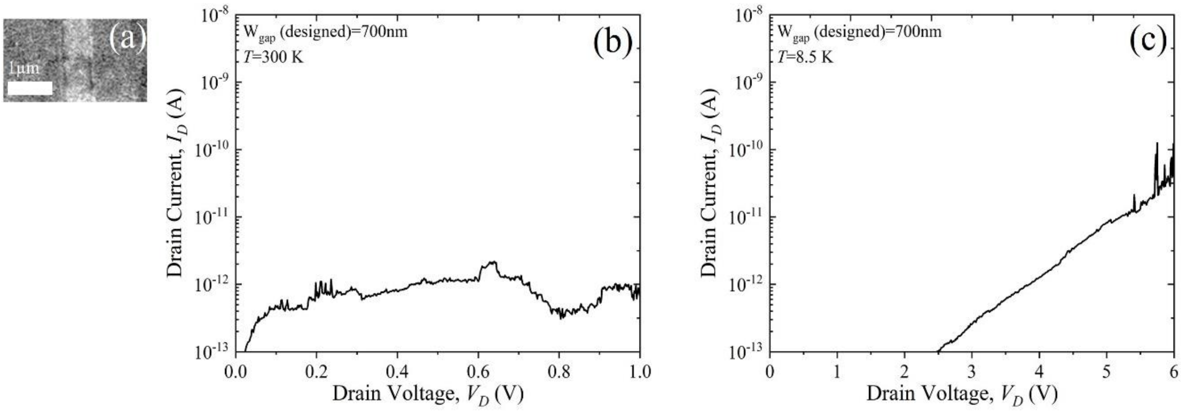

Figure S4. (a) FE-SEM image of a CNT bridge network deposited by inkjet printing in a gap (designed gap = 700 nm) between Al source and drain electrodes. (b) Room-temperature I_D–V_D characteristics for the same device. (c) Low-temperature (T = 8.5 K) I_D–V_D characteristics for the same device. Characteristics are measured in different V_D ranges as the onset voltage is largely shifted at low temperature.

We show here (Figs. S3a and S4a) a few additional examples of CNT-networks bridging source-drain gaps of different dimensions. I-V characteristics shown here for each device were measured in different ranges and for different temperatures, illustrating the critical effect on the conductivity. As a function of temperature, a shift of the onset to high voltages is observed as temperature is reduced, as shown by comparatively analyzing the I_D–V_D characteristics in Figs. S3b-c and, respectively, in Figs. S4b-c.

Misalignment of CNT network and proof-of-concept manipulation:

As we can see in Fig. S5, when the gap is prepared with a lateral size of a few µm (with a so-called trapezoidal gap), it is likely to miss the alignment of the CNT-networks within the shortest-gap area. Some CNT-networks are found below the targeted location (as marked in Fig. S5a), although still contributing as paths to transport the current (as observed by the current, I_D, in Fig. S5b).

Despite the current flowing, it can also be noticed that the current level is relatively low (up to a few tens of pA even for V_D = 6 V). In order to control the arrangement of such CNT-networks, one possible approach is to employ a high-speed AFM nano-manipulator coupled with a haptic device [1].

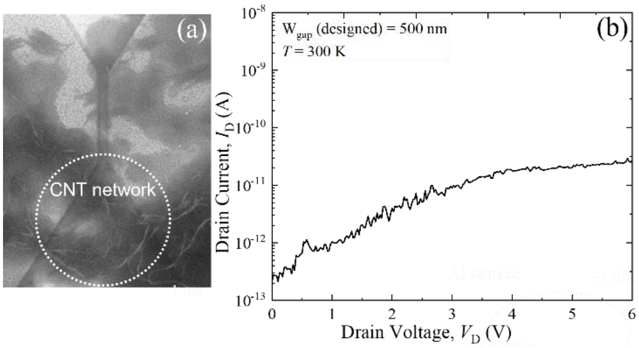

Figure S5. (a) FE-SEM image of a CNT bridge network deposited by inkjet printing in a trapezoidal gap (lateral size of 3 µm) between Al source and drain electrodes. The network is deposited not in the nearest gap, but below, illustrating the present alignment capabilities. (b) Room-temperature I_D–V_D characteristics for the same device, with a designed trapezoidal gap of 500 nm.

Figure S6 shows basic demonstrations of the modification of CNT networks deposited from NMP-solution

on a glass surface (for ease of test) when the AFM tip was moved (dragged) over the area marked by a dotted circle, at specific locations. For this study, an SPI4000 AFM system was used, with an HQ:NSC15 cantilever with a resonant frequency of 325 kHz and a force constant of 40 N/m. All measurements were done in tapping mode, in air, with a tip-to-sample distance of approximately 1 nm.

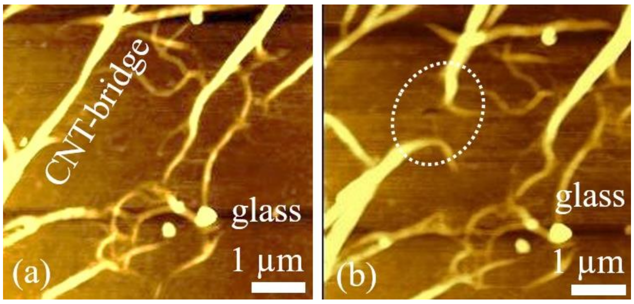

Figure S6. AFM images of a CNT-network deposited on a glass substrate from NMP solution before (a) and after (b) AFM manipulation (disconnection) of a CNT-bridge (in the area marked by the dotted circle).

The effects of the nanomanipulation can be observed by comparing the images in: (a) before AFM manipulation and (b) after AFM manipulation.

In this test, it can be clearly shown how the AFM manipulation allows the disconnection (interruption) of a CNT-bridge (as labeled in Fig. S6a), breaking the pathway in the region marked by a dotted circle (in Fig. S6b). It is likely that this is due to the separation of adjacent CNTs or CNT-bundles weakly coupled within the CNT-bridge. Further manipulation is feasible, although additional study is necessary.

Although the CNT-network shown here is obtained from NMP solution on a glass substrate (for simplicity of the preliminary test by AFM), it is comparable with the network shown in Fig. S5a. As such, AFM manipulation demonstrated here can be considered a proof of concept for application to the actual devices, potentially allowing the adjustment of conductance by aligning and optimizing the CNT networks. A proof-of-concept demonstration for CNT-networks deposited on SiO_2 is shown in the paper, Fig. 4.
